# Five-Year Evolution of the Microbiological Landscape of Diabetic Foot Infections (2021–2025): Rising Polymicrobial Gram-Negative Predominance and Emergence of Resistant Enterobacterales at a Single Large-Volume Outpatient Centre

**DOI:** 10.3390/antibiotics15080723

**Published:** 2026-07-24

**Authors:** Magdalena Maj, Leszek Czupryniak

**Affiliations:** 1 Dr Maj—Diabetic Foot Prevention and Treatment Center, 62-002 Poznań, Poland; 2 Department of Diabetology and Internal Medicine, Medical University of Warsaw, 02-097 Warsaw, Poland; leszek.czupryniak@wum.edu.pl

**Keywords:** diabetic foot infection, antimicrobial resistance, methicillin-resistant *Staphylococcus aureus*, Enterobacterales, *Proteus mirabilis*, MALDI-TOF, EUCAST, antimicrobial stewardship, Poland, surveillance

## Abstract

Background/Objectives: Diabetic foot infections (DFI) drive most non-traumatic lower-limb amputations, and empirical regimens must track local microbiology, which is itself evolving under broad-spectrum antibiotic pressure. Methods: We retrospectively analysed all consecutive bacterial cultures from clinically infected diabetic foot lesions at a single Polish outpatient centre over 2021–2025. Isolates were identified by MALDI-TOF mass spectrometry and susceptibility was interpreted under contemporaneous EUCAST breakpoints (v12.0–v15.0). Results: A total of 274 cultures yielded 443 isolates (polymicrobial index 1.62). *Staphylococcus aureus* remained the most common pathogen but fell from 55.0% to 38.9% of cultures, while Enterobacterales rose from 36.1% to 47.7% of all isolates. *Proteus mirabilis* and *Streptococcus agalactiae* emerged as major contributors, and *Enterobacter hormaechei* showed an apparent increase (the apparent rise in *E. hormaechei* reflects, at least in part, improved species-level identification rather than a true increase in incidence). Methicillin-resistant *S. aureus* was first documented in 2025 (6/38; 15.8%). *P. mirabilis* showed deteriorating activity against trimethoprim–sulfamethoxazole and ciprofloxacin, the first meropenem-resistant isolate appeared in 2025, and two carbapenem-non-susceptible Enterobacterales were also detected (2/18 carbapenem-tested isolates in 2025; 11%). Conclusions: The DFI profile has shifted from an *S. aureus*-dominated Gram-positive ecology toward a polymicrobial, increasingly Gram-negative one, with the first documentation of MRSA (on reintroduction of routine oxacillin testing) and of carbapenem-non-susceptible Enterobacterales. Empirical policy for moderate-to-severe DFI should reliably cover Enterobacterales and methicillin-resistant Gram-positives while preserving carbapenems and glycopeptides through active stewardship.

## 1. Introduction

Diabetic foot infection (DFI) is the single most common indication for hospitalisation in people living with diabetes and underlies the majority of non-traumatic lower-limb amputations worldwide [1]. Successful management depends on early, appropriately targeted antimicrobial therapy combined with adequate debridement and vascular intervention where indicated. Because clinical signs are often blunted by neuropathy and immunometabolic dysfunction, empirical antibiotic choices remain anchored to the locally prevailing microbiological flora and its resistance profile rather than to the bedside picture alone [2,3,4,5].

The 2023 intersociety guideline of the International Working Group on the Diabetic Foot (IWGDF) and the Infectious Diseases Society of America (IDSA) recommends *Staphylococcus aureus* and β-haemolytic streptococci as the default empirical targets in mild infection, with the addition of Gram-negative and anaerobic cover in moderate-to-severe disease, ischaemia or prior antibiotic exposure [2]. A pooled meta-analysis of 22,198 isolates from 16,159 patients across 112 studies confirmed *S. aureus* as the single most prevalent pathogen worldwide, of which 18.0% (95% CI 13.8–22.6%) were methicillin-resistant; *Pseudomonas* spp., *Escherichia coli* and *Enterococcus* spp. were also consistently identified, and Gram-negative predominance was associated with lower Gross National Income [3]. Contemporary systematic reviews covering studies published between 2014 and 2024 have confirmed both this composition and a steady deterioration of in vitro activity for several first-line oral agents, with rising resistance to amoxicillin–clavulanate, trimethoprim–sulfamethoxazole and ciprofloxacin [4,5].

Regional data are now diverging from the historical Gram-positive paradigm. A Polish single-centre series from 2015 to 2016 reported that 77.1% of isolated bacteria from DFI were Gram-negative [6], a finding broadly mirrored in a 1199-patient chronic-wound dataset from a Polish wound-care centre [7]. Surveillance from northeastern Italy reported MRSA in 27.1% of *S. aureus*-positive DFI cases and substantial fluoroquinolone resistance among *P. aeruginosa* [8]. Investigations from southern Poland (Kraków) and Gdańsk have documented MRSA in 10–15% of DFI *S. aureus* isolates, with MRSA carriage associated with a higher incidence of underlying osteitis [9,10]. From low- and middle-income settings, recent cross-sectional data from the West Bank, Palestine, show Gram-negative predominance (58.8%) and substantial multidrug resistance among DFI isolates [11], while Indian tertiary-care data document 50% ESBL prevalence among *E. coli*/*Klebsiella*/*Proteus* from DFI and a 39% carbapenem-resistant Enterobacterales (CRE) rate [12].

The microbiology laboratory itself has been a moving target during this period. Routine deployment of matrix-assisted laser desorption/ionisation time-of-flight mass spectrometry (MALDI-TOF MS) has expanded the resolution at which clinical isolates are identified, but the technique has limitations within closely related taxa. The *Enterobacter cloacae* complex (ECC), in particular, is poorly resolved by default Bruker Biotyper libraries: *E. hormaechei*—the ECC species most strongly associated with acquired multidrug resistance and carbapenemase production—was historically reported as *E. cloacae* on standard spectra, with correct species-level assignment achievable in only 20–25% of cases without dedicated reference libraries or machine-learning–based algorithms [13,14]. The apparent increase in *E. hormaechei* in clinical datasets therefore reflects, at least in part, a methodological shift. In parallel, the EUCAST framework has continued to evolve, with revised breakpoints for piperacillin–tazobactam, cefiderocol, ciprofloxacin (staphylococci), and the addition of the ‘I = susceptible at increased exposure’ category, all bearing directly on how resistance trends are read across surveillance years [15,16].

Against this background, regional surveillance data are critical to keep empirical algorithms current. We undertook a five-year retrospective audit of all bacterial cultures obtained from diabetic foot lesions at our institution between 2021 and 2025, with the dual aim of characterising the temporal evolution of the microbiological profile and quantifying the corresponding shifts in antimicrobial resistance—while explicitly accounting for the laboratory- and breakpoint-level changes that occurred during the same period.

## 2. Results

### 2.1. Culture Volume and Overall Microbial Yield

Across the five-year audit period, 274 diabetic foot cultures yielded 443 bacterial isolates (Table 1). Annual culture volume rose from 20 in 2021 to 131 in 2025—a 6.6-fold increase that paralleled the development of a dedicated diabetic foot service; as discussed below, this growth may also reflect changes in referral pattern and case mix, so the accompanying compositional changes cannot be attributed to a true shift in the underlying epidemiology on the basis of these data alone. The polymicrobial index remained consistently above unity throughout the audit (maximum 1.80 in 2021, minimum 1.46 in 2024, 1.68 in 2025), confirming that polymicrobial infection is the rule rather than the exception in this cohort. The number of distinct species recovered per year rose from 15 in 2021 to 38 in 2025, reflecting both improved diagnostic resolution under routine MALDI-TOF and a genuine broadening of the bacterial flora.

### 2.2. Evolution of Pathogen Composition

**Figure 1:**
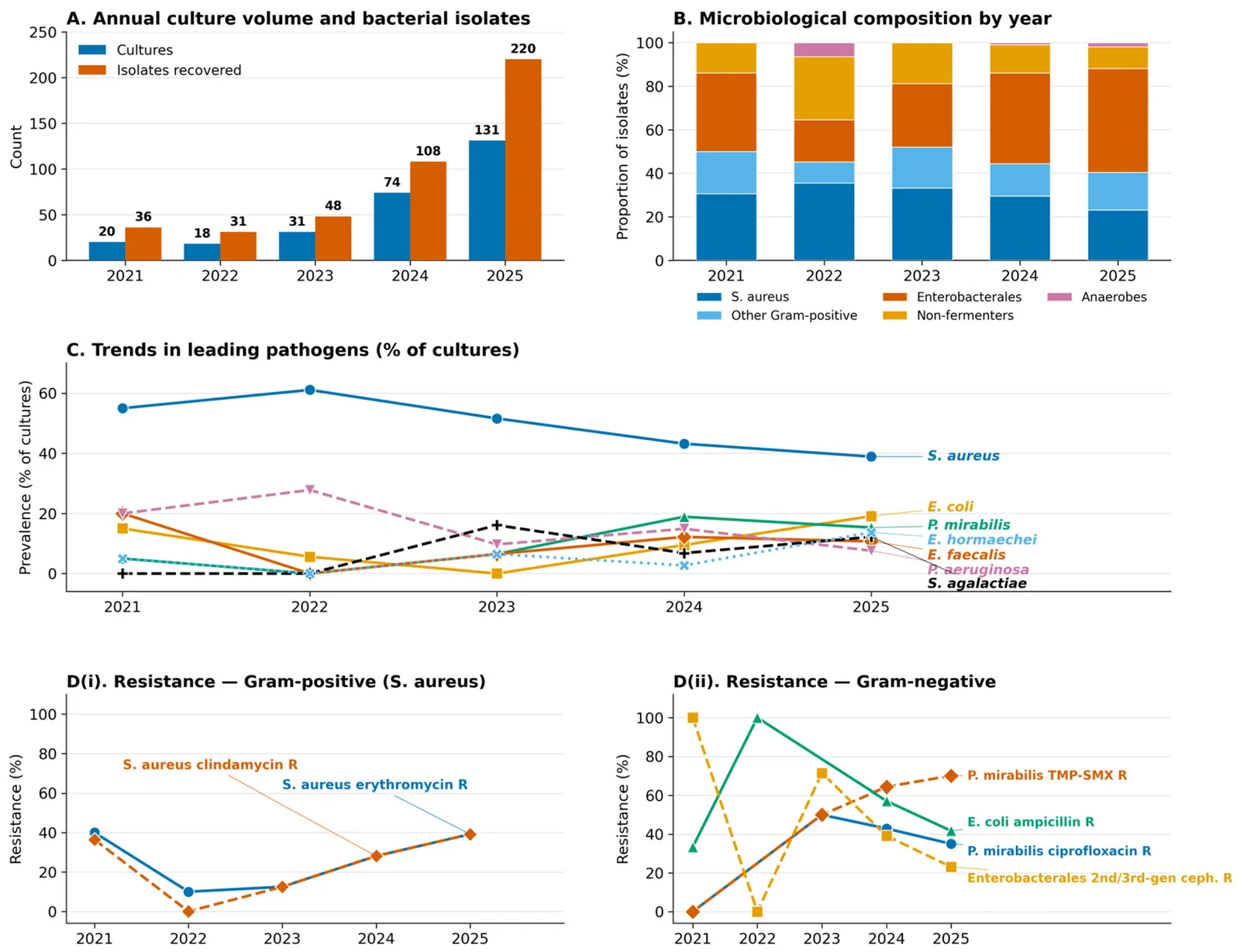
Evolution of diabetic foot infection microbiology, 2021–2025. (**A**) Annual numbers of cultures (left bars) and bacterial isolates recovered (right bars). (**B**) Stacked composition of bacterial isolates by major group, expressed as percentage of total isolates per year. (**C**) Annual prevalence of leading pathogens (percentage of cultures yielding the organism). (**D**) Resistance trajectories of selected clinically important antibiotics. *S. aureus* erythromycin/clindamycin resistance shown together as a surrogate of inducible/constitutive MLSB phenotype. The 100% point for Enterobacterales/cefuroxime in 2021 reflects only two tested isolates. Abbreviations: GP, Gram-positive; non-ferm., non-fermenters; MLSB, macrolide–lincosamide–streptogramin B; TMP-SMX, trimethoprim–sulfamethoxazole; ceph., cephalosporin. For legibility, panels (**C**,**D**) use a colour-blind-safe palette with distinct markers and directly labelled curves, and the resistance trajectories in panel (**D**) are separated into Gram-positive [panel (**D(i)**)] and Gram-negative [panel (**D(ii)**)] agents.

Staphylococcus aureus was the dominant pathogen throughout the period but its relative prevalence fell from 55.0% of cultures in 2021 to 38.9% in 2025 (Table 2, Figure 1C). This decline was statistically significant on the Cochran–Armitage test for trend (z = −2.15, *p* = 0.031). The relative weight of Enterobacterales rose in parallel, accounting for 36.1% of all isolates in 2021 and 47.7% in 2025 (Figure 1B). The rising Enterobacterales share was significant (trend z = 3.05, *p* = 0.002), whereas the parallel fall in the Gram-positive share did not reach significance (z = −1.58, *p* = 0.11). The most striking compositional shifts were the increases in Proteus mirabilis (5.0% → 15.3% of cultures), Enterobacter hormaechei (5.0% → 13.7%) and Streptococcus agalactiae (absent in 2021–2022, 12.2% in 2025). Escherichia coli showed a U-shaped trajectory, fluctuating between 0% and 9.5% during 2022–2024 before rebounding sharply to 19.1% in 2025. Across the full period the rise in *P. mirabilis* reached significance (trend z = 2.08, *p* = 0.038), and that of E. coli was of borderline significance (z = 1.96, *p* = 0.050); both should be read with caution given the year-on-year variation in denominators. Pseudomonas aeruginosa, a classical pathogen of moist or ischaemic wounds, declined progressively from 20 to 28% in the early years to 7.6% in 2025. Enterobacter cloacae, common in 2021–2023, was not recovered in 2024–2025, consistent with displacement by *E. hormaechei* following improved species-level resolution of the historic E. cloacae complex—an effect documented in multiple recent MALDI-TOF performance studies [13,14].

Anaerobes were recovered in only six specimens across the entire period (1.4% of all isolates), a yield substantially lower than that reported in deep-tissue series elsewhere and consistent with limitations of contemporary swab-based sampling for fastidious organisms. Of note, tissues were sampled for anaerobes only when it was clinically indicated. Because this low yield is most likely an artefact of sampling and transport rather than a true absence of anaerobes, empirical anaerobic cover (e.g., metronidazole, or an agent with intrinsic anaerobic activity such as piperacillin–tazobactam) should still be considered for moderate-to-severe, deep, necrotic or ischaemic DFI irrespective of culture recovery.

### 2.3. Antimicrobial Resistance Trends

*Staphylococcus aureus*. Routine oxacillin (cloxacillin) testing was reintroduced only in 2025, when methicillin-resistant *S. aureus* (MRSA) was detected in six of 38 tested isolates (15.8%, 95% CI 6.0–31.3%). Because oxacillin was not routinely tested before 2025, this figure represents a single-year point prevalence rather than a documented temporal increase, and the prior-year MRSA burden cannot be estimated from these data. Macrolide–lincosamide (MLSB) resistance—captured by paired erythromycin and clindamycin resistance—rose from 12% (2/16) in 2023 to 39% (20/51) in 2025 (Table 3, Figure 1D); the increase across 2022–2025 was significant on the Cochran–Armitage trend test (z = 2.49, *p* = 0.013). Trimethoprim–sulfamethoxazole retained complete in vitro activity throughout the period (0/118 resistant), and gentamicin resistance remained below 5%. Gentamicin resistance developed in patients treated with local gentamicin application (e.g., Stimulan G) for prolonged periods often exceeding 6 weeks. Fusidic acid resistance, which had risen briefly in 2023, returned to single digits by 2025.

Enterobacterales. Among Enterobacterales, ampicillin and amoxicillin resistance remained near-universal where intrinsic. Acquired ampicillin resistance in *E. coli* was 42% in 2025, and in *P. mirabilis* it was 60%. Resistance to second- and third-generation cephalosporins (cefuroxime/cefotaxime), used as a surrogate for ESBL production, peaked at 69% of tested Enterobacterales in 2023 and declined to 36% in 2024 and 24% in 2025; whether this reflects genuine epidemiological change, antimicrobial-stewardship pressure or shifts in the species mix cannot be resolved without genotypic data. Ciprofloxacin resistance in *E. coli* fell from 29% (2024) to 12% (2025), but in *P. mirabilis* remained between 35% and 43%. Trimethoprim–sulfamethoxazole, often selected as oral step-down therapy, lost activity progressively against *P. mirabilis* (50% R in 2023, 64% in 2024, 70% in 2025). Two carbapenem-non-susceptible Enterobacterales were detected in 2025 (one *Proteus mirabilis* resistant to meropenem and one carbapenem-intermediate isolate), corresponding to 11% (2/18) of carbapenem-tested isolates—the first such finding in the audit period.

*Pseudomonas aeruginosa*. Anti-pseudomonal activity remained generally preserved. Ciprofloxacin resistance was 0% through 2023 but reached 10–18% in 2024–2025. Piperacillin–tazobactam resistance rose modestly from 0% in 2022–2023 to 20% in 2025 (2/10), although this should be interpreted in the light of the EUCAST piperacillin–tazobactam breakpoint revision that occurred during the audit period [16]. One of the small number of meropenem-tested *P. aeruginosa* isolates was resistant in 2025; given the very small denominator (n = 1), this finding requires confirmation in subsequent surveillance cycles.

Enterococci and streptococci. *Enterococcus faecalis* retained universal in vitro susceptibility to ampicillin (0/29 tested R) and to the glycopeptides vancomycin and teicoplanin (0/23 tested R). No vancomycin-resistant enterococci, linezolid-resistant Gram-positives, or penicillin-resistant β-haemolytic streptococci were identified during the audit period.

### 2.4. Clinical Snapshot of Enterococcus faecalis-Positive Infections

To provide a clinical anchor for the microbiological findings, and in response to reviewer requests for clinical correlation, we extracted summary clinical data for all patients whose cultures yielded *Enterococcus faecalis* (n = 29; 4, 0, 2, 9 and 14 patients in 2021–2025, respectively), the organism chosen because its case count spans the full audit period and its susceptibility profile was stable. The cohort was predominantly male (23/29) with a median age of 66 years (range 50–85); peripheral arterial disease (ankle–brachial index <0.9) was present in 19 of 28 patients with a recorded index, and infection was polymicrobial in 23 of 28 (Table 4). All isolates were ampicillin-susceptible and, where tested, susceptible to vancomycin and teicoplanin, consistent with Table 3. Fifteen patients healed (median time to healing 15 weeks, range 5–30), eight underwent minor or major amputation, two died (one after amputation), one remained on treatment and three were lost to follow-up. Adverse outcomes (amputation or death) were concentrated among patients with peripheral arterial disease (8/19 with ABI < 0.9 vs. 1/9 with ABI ≥ 0.9) and a higher comorbidity burden (5/15 with ≥3 comorbidities vs. 1/8 with ≤1), whereas patients without significant comorbidity and with fewer co-isolated species generally healed. These descriptive associations are based on a single-organism subgroup with small numbers and incomplete denominators; they are hypothesis-generating and do not establish causation, but they indicate that the cohort spans the expected clinical spectrum of diabetic foot infection and that case mix, rather than improving, broadened as culture volume grew. Patient-level data are provided in Supplementary Table S1.

## 3. Discussion

Our five-year audit documents three convergent changes in the microbiology of diabetic foot infection at this Polish centre: a relative decline of *S. aureus* dominance in favour of a more diverse, polymicrobial flora; a clear emergence of *Proteus mirabilis* and *Streptococcus agalactiae*, together with an apparent increase in *Enterobacter hormaechei*, as quantitatively important pathogens; and the parallel appearance of clinically meaningful resistance phenotypes—including MRSA and the first carbapenem-non-susceptible Enterobacterales—against a background of preserved glycopeptide and aminoglycoside activity.

The relative ascendancy of Gram-negative organisms in our cohort fits a broader regional pattern. A Polish single-centre cohort from 2015 to 2016 already reported 77.1% Gram-negative isolation in DFI [6], and a Polish chronic-wound dataset spanning 2013–2021 confirmed *S. aureus* (14.3% of which were MRSA) and *E. faecalis* as the dominant Gram-positives within a heavily polymicrobial flora [7]. The 18.0% pooled MRSA prevalence from the global meta-analysis [3] and the 27.1% rate from northeastern Italy [8] bracket our own 2025 estimate of 15.8% (6/38). The 10.3% rate from Kraków [9] and the 15.4% rate from Gdańsk [10] sit immediately adjacent. Our finding therefore does not represent an outlier so much as the first reliable measurement in our centre, made possible by the reintroduction of routine oxacillin testing in 2025; the apparent emergence is in part a surveillance artefact and would not justify, on its own, a switch to empirical anti-MRSA cover in mild disease.

The substantial rise in *E. hormaechei* deserves cautious interpretation. The *E. cloacae* complex is poorly resolved by default MALDI-TOF reference libraries; the Bruker Biotyper database correctly assigns *E. hormaechei* at the species level in approximately 20% of cases, rising to 92–97% only when extended online libraries (such as the MSI database) or supervised machine-learning algorithms are applied [13,14]. A proportion of what we now identify as *E. hormaechei* was almost certainly reported as *E. cloacae* or simply as ECC in the early years of the audit. The fall in *E. cloacae* to zero in 2024–2025, mirrored by the rise in *E. hormaechei*, is consistent with this reclassification. A substantial proportion of the apparent rise in *E. hormaechei* therefore likely represents improved species-level resolution rather than a true increase in incidence, and the organism should not be described as a newly emergent pathogen on the basis of these data alone. The clinical implications nonetheless remain relevant: *E. hormaechei* carries a higher prevalence of third-generation cephalosporin resistance than other ECC members and is the principal ECC species associated with carbapenemase acquisition (notably KPC and OXA-48 enzymes on ST114, ST90 and ST93 backgrounds) [13].

The emergence of *Streptococcus agalactiae* (group B Streptococcus, GBS) from 0% to 12.2% of cultures is concordant with several recent reports of rising GBS incidence in non-pregnant adults with diabetes, in whom it can cause severe deep soft-tissue infection, tenosynovitis and rare streptococcal toxic-shock-like syndrome [17,18]. Although GBS remained universally susceptible to penicillin in our data, reduced-susceptibility phenotypes have been described and warrant continued monitoring [19].

The deterioration of oral options for *P. mirabilis* is the most clinically pressing finding. TMP-SMX resistance rose from 50% (1/2) in 2023 to 64% (9/14) in 2024 and 70% (14/20) in 2025, and ciprofloxacin resistance settled at 35–43% (6/14 in 2024 and 7/20 in 2025), with the first meropenem-resistant isolate identified in 2025 (one of only two isolates tested that year; the trend in TMP-SMX resistance across 2023–2025 did not reach significance, z = 0.59, *p* = 0.56, reflecting the small early denominators). These rates exceed those reported in older Canadian/UK/US series (60–90% ciprofloxacin susceptibility) [20] and are now approaching the resistance profiles described in clonal NDM-producing *P. mirabilis* outbreaks from Tunisian and Chinese diabetic foot cohorts [21,22]. The implication for prescribing is direct: TMP-SMX and ciprofloxacin should no longer be considered reliable empirical oral options for *P. mirabilis* in this setting, and unexplained clinical failure on these agents warrants prompt resampling and broadening of cover.

The apparent decline in ESBL-phenotype prevalence among Enterobacterales between 2023 and 2025 is encouraging but should be interpreted cautiously. The denominator changed substantially over the period, surrogate phenotypic markers do not reliably discriminate ESBL from AmpC or porin-loss mechanisms, and the appearance of two carbapenem-non-susceptible Enterobacterales in 2025 raises the possibility of clonal selection that the cefuroxime-based screen may not capture. In the Indian tertiary-care series of Das and colleagues, half of all *E. coli*/*K. pneumoniae*/*P. mirabilis* from DFI were ESBL producers and 39% of all Gram-negative isolates were CRE [12], and broadly similar findings have been reported from a recent cross-sectional study in a low- to middle-income setting [11]. Routine confirmatory testing for ESBL, AmpC and carbapenemase genes—ideally by molecular methods, with whole-genome sequencing where outbreaks are suspected [23]—is warranted going forward.

Several limitations bear on the strength of the inferences that can be drawn. The 6.6-fold growth in culture volume across the period limits direct year-on-year comparison: the denominators in 2021 and 2022 are too small for inferential statistics, and observed proportional changes in those years must be read as descriptive only. The broadening of the antibiotic panel from 19 agents in 2021 to 32 agents in 2025 means that some resistance trends reflect newly introduced testing rather than newly emerging resistance, a limitation we have tried to mitigate by reporting resistance only for isolate–antibiotic pairs actually tested. The EUCAST framework itself changed across the audit, notably for piperacillin–tazobactam, ciprofloxacin against staphylococci, and cefiderocol [15,16], which compresses the comparability of resistance estimates between calendar years. Other limitations include the single-centre design, the limited availability of linked clinical metadata for the full cohort (PEDIS/IDSA grade, ulcer chronicity, glycaemic control and prior antibiotic exposure were not systematically captured for every isolate, although a clinical snapshot is provided for one representative organism in Section 2.4), the variable use of swab versus deep-tissue sampling, the under-representation of anaerobes consistent with that sampling mix, and the absence of genotypic resistance data.

Our findings sit within a wider European trend recently captured in a multicentre cohort spanning two decades and five academic centres (Geneva, Zurich, Las Palmas, Barcelona, Istanbul), which documented a significant increase in Enterobacteriaceae as causative pathogens of diabetic foot osteomyelitis, particularly between 2016 and 2019 [24]. A contemporary Turkish single-centre series of 262 patients (2021–2022) using EUCAST methodology reported 57.6% Gram-negative isolates, 51.3% methicillin-resistant *Staphylococcus* spp. and 66.7% ESBL-positive *E. coli*—substantially higher resistance burdens than ours but the same compositional shift toward polymicrobial Gram-negative infection [25]. A Brazilian tertiary-care series reported a comparable Gram-negative predominance and reinforced the case for centre-specific empirical algorithms [26]. Sequencing-based microbiome analyses from a multicentre Spanish cohort have further linked worse clinical outcomes to polymicrobial, Gram-negative-enriched flora [27], underlining the value of expanding diagnostics beyond conventional culture. Translated into a practical bedside algorithm at our centre, and pending confirmation with larger multicentre datasets and linked clinical outcomes, the present data support consideration of continued use of an oral agent active against *S. aureus* and β-haemolytic streptococci (e.g., cephalexin or amoxicillin–clavulanate) for mild DFI; an Enterobacterales-active regimen with anti-MRSA cover (e.g., piperacillin–tazobactam, or a third-generation cephalosporin combined with clindamycin or a glycopeptide) for moderate-to-severe DFI in patients without recent antipseudomonal exposure; and reservation of carbapenems for severe infection with recent ESBL exposure or sepsis, pending culture-directed de-escalation in line with IWGDF/IDSA 2023 stewardship principles [2]. These suggestions are centre-specific and provisional: they describe a local empirical framework derived from surveillance data without linked outcome validation and should not be generalised to other settings without confirmation in larger, multicentre cohorts with prospective clinical follow-up.

Despite these caveats, the temporal signal is consistent and clinically coherent, and it converges with the picture emerging from contemporary Polish, European and global surveillance. Diabetic foot infections at our centre have evolved from a predominantly Gram-positive, *S. aureus*-driven syndrome toward a polymicrobial, increasingly Gram-negative ecology, and clinically important resistance phenotypes have begun to appear. Continued local surveillance, integration of molecular diagnostics for resistance mechanisms, and prospective coupling of microbiological data to outcomes will be essential to keep empirical regimens calibrated to a moving target.

## 4. Materials and Methods

### 4.1. Study Design and Setting

This was a retrospective observational study of consecutive bacterial cultures obtained from clinically infected diabetic foot lesions at a single outpatient diabetes foot centre in Poland over five consecutive calendar years (1 January 2021 to 31 December 2025). Infection was diagnosed clinically by the treating team in accordance with the IWGDF/IDSA 2023 framework [2], defined as the presence of at least two local signs of inflammation (erythema, warmth, induration/swelling, tenderness or pain, or purulent secretion) or systemic signs of infection in the context of a foot ulcer or wound, and culture was performed at the discretion of that team. The microbiology laboratory dataset was de-identified prior to analysis. Because the analysis used only routinely collected, anonymised laboratory data, formal ethics-committee approval was waived in line with institutional policy.

### 4.2. Specimen Collection and Processing

Specimens were obtained from infected ulcers by tissue curettage, deep-tissue biopsy or aspiration of purulent collections; superficial swabs were discouraged but accepted when no other material was available, consistent with current guideline practice [2]. Samples were processed within 2 h of collection on standard aerobic and selective enrichment media; anaerobic culture was performed on tissue and aspirate specimens. Anaerobic culture was therefore requested only for deep-tissue and aspirate specimens (not for swabs); such specimens were transported in an anaerobic transport medium and processed promptly, and the low anaerobic yield should be read in the light of this selective, sampling-dependent ascertainment. Where the same species was isolated more than once from the same lesion in the same patient within a 14-day window, only the first isolate was retained, so that polymicrobial indices and resistance proportions were calculated on de-duplicated, episode-level data. Bacterial identification was carried out by matrix-assisted laser desorption/ionisation time-of-flight mass spectrometry (MALDI-TOF MS) on a Bruker Biotyper platform. In brief, intact bacterial colonies are overlaid with an energy-absorbing matrix; a pulsed laser desorbs and ionises predominantly ribosomal proteins, which are then separated by their time of flight through a vacuum tube, generating a characteristic mass spectrum that is matched against a reference database to yield species-level identification. For ECC isolates, species-level resolution within the complex relied on the manufacturer’s library; we acknowledge that this approach may misclassify a proportion of *E. hormaechei* isolates as *E. cloacae* in earlier years of the audit period [13,14].

### 4.3. Antimicrobial Susceptibility Testing

Antimicrobial susceptibility was determined using the disc-diffusion method and/or automated broth microdilution and interpreted according to the contemporary EUCAST breakpoint tables in force each calendar year (v12.0 in 2022 through v15.0 in 2025) [15]. Results were reported using the revised EUCAST categories: susceptible at standard exposure (S; recorded as W in the source dataset, Polish Wrażliwy), susceptible at increased exposure (I; WZE, Wrażliwy w Zwiększonej Ekspozycji) and resistant (R; O, Oporny). The antibiotic panel expanded over the study period from 19 agents in 2021 to 32 agents in 2025, including the addition of newer agents such as ceftazidime–avibactam and linezolid in the final year; analyses of resistance rates are therefore confined to isolate–antibiotic pairs for which testing was performed in the index year. Revisions to the piperacillin–tazobactam breakpoint for Enterobacterales (with the introduction of an I/SDD category) occurred during the audit period and may have shifted the proportion of isolates reported as fully susceptible from 2022 onward [16].

### 4.4. Definitions and Analysis

The polymicrobial index was defined as the mean number of distinct bacterial isolates per culture. Pathogens were classified as Gram-positive cocci, Enterobacterales, non-fermentative Gram-negative bacilli, or anaerobes. Methicillin-resistant *S. aureus* (MRSA) was inferred from oxacillin/cloxacillin (kloksacylina) resistance. Resistance of Enterobacterales to second- and third-generation cephalosporins (cefuroxime and cefotaxime) served as a surrogate marker of potential ESBL production; this was not confirmed by genotypic testing [11,12]. Multidrug resistance was defined according to the Magiorakos consensus criteria (acquired non-susceptibility to ≥1 agent in ≥3 antimicrobial classes) [28]. Resistance rates were expressed as the proportion of tested isolates within a given species and year. Temporal trends across the five calendar years were assessed with the Cochran–Armitage test for trend in proportions, using the calendar year as the ordinal score; two-sided *p*-values < 0.05 were considered statistically significant. Because the 2021 and 2022 denominators were small, trend testing was applied principally to organism-group and leading-pathogen proportions, for which the pooled denominators were adequate, and a sensitivity analysis restricted to the higher-volume years (2023–2025) was performed for selected resistance phenotypes; results from cells with fewer than five tested isolates are reported as raw fractions and were not subjected to inferential testing. Where denominators permit, 95% confidence intervals (Wilson method) are provided. Analyses were performed in Python 3.11 (pandas 2.2, SciPy 1.13, statsmodels 0.14, matplotlib 3.9).

## 5. Conclusions

Between 2021 and 2025 the microbiological profile of diabetic foot infection at this Polish centre shifted measurably toward polymicrobial Gram-negative infection, with the first documentation of MRSA following reintroduction of routine oxacillin testing and of the first carbapenem-non-susceptible Enterobacterales. Findings are concordant with recent regional surveillance and with the IWGDF/IDSA 2023 framework. Empirical antibiotic policy for moderate-to-severe DFI in this setting should now provide reliable cover for Enterobacterales and methicillin-resistant Gram-positives, while preserving carbapenems and glycopeptides through active stewardship and continued local surveillance.

## Figures and Tables

**Table 1:** Annual specimen volume, isolate yield and microbiological composition, 2021–2025. Group counts are isolates; percentages are of total isolates per year.

Total	2025	2024	2023	2022	2021	Variable
274	131	74	31	18	20	Cultures, n
443	220	108	48	31	36	Isolates, n
1.62	1.68	1.46	1.55	1.72	1.8	Isolates per culture
—	38	22	21	13	15	Distinct species, n
186 (42.0%)	87 (39.5%)	42 (38.9%)	25 (52.1%)	14 (45.2%)	18 (50.0%)	Gram-positive, n (%)
181 (40.9%)	105 (47.7%)	44 (40.7%)	13 (27.1%)	6 (19.4%)	13 (36.1%)	Enterobacterales, n (%)
56 (12.6%)	22 (10.0%)	14 (13.0%)	6 (12.5%)	9 (29.0%)	5 (13.9%)	Non-fermenters, n (%)
6 (1.4%)	4 (1.8%)	0 (0%)	0 (0%)	2 (6.5%)	0 (0%)	Anaerobes, n (%)

**Table 2:** Prevalence of leading bacterial isolates in diabetic foot cultures, by year (% of cultures yielding the organism).

2025 (n = 131)	2024 (n = 74)	2023 (n = 31)	2022 (n = 18)	2021 (n = 20)	Organism
51 (38.9%)	32 (43.2%)	16 (51.6%)	11 (61.1%)	11 (55.0%)	*Staphylococcus aureus*
25 (19.1%)	7 (9.5%)	0 (0%)	1 (5.6%)	3 (15.0%)	*Escherichia coli*
20 (15.3%)	14 (18.9%)	2 (6.5%)	0 (0%)	1 (5.0%)	*Proteus mirabilis*
18 (13.7%)	2 (2.7%)	2 (6.5%)	0 (0%)	1 (5.0%)	*Enterobacter hormaechei*
16 (12.2%)	5 (6.8%)	5 (16.1%)	0 (0%)	0 (0%)	*Streptococcus agalactiae*
14 (10.7%)	9 (12.2%)	2 (6.5%)	0 (0%)	4 (20.0%)	*Enterococcus faecalis*
10 (7.6%)	3 (4.1%)	2 (6.5%)	2 (11.1%)	2 (10.0%)	*Klebsiella oxytoca*
10 (7.6%)	11 (14.9%)	3 (9.7%)	5 (27.8%)	4 (20.0%)	*Pseudomonas aeruginosa*
7 (5.3%)	0 (0%)	0 (0%)	1 (5.6%)	1 (5.0%)	*Morganella morganii*
7 (5.3%)	2 (2.7%)	1 (3.2%)	0 (0%)	0 (0%)	*Klebsiella pneumoniae*
3 (2.3%)	4 (5.4%)	1 (3.2%)	0 (0%)	1 (5.0%)	*Proteus vulgaris*
3 (2.3%)	1 (1.4%)	2 (6.5%)	0 (0%)	0 (0%)	*Acinetobacter baumannii*
0 (0%)	0 (0%)	2 (6.5%)	1 (5.6%)	3 (15.0%)	*Enterobacter cloacae*

**Table 3:** Antimicrobial resistance trends in selected pathogens, 2021–2025. Cells show the proportion of tested isolates classified as resistant (R%) with the number tested in parentheses. NT = not tested or fewer than two isolates available.

2025	2024	2023	2022	2021	Pathogen/Antibiotic
16% (38)	NT	NT	0% (7)	NT	*S. aureus*—Oxacillin (MRSA)
39% (51)	28% (32)	12% (16)	10% (10)	40% (10)	*S. aureus*—Erythromycin
39% (51)	28% (32)	12% (16)	0% (10)	36% (11)	*S. aureus*—Clindamycin
8% (12)	6% (31)	17% (12)	40% (5)	9% (11)	*S. aureus*—Ciprofloxacin
16% (50)	19% (32)	6% (16)	50% (2)	27% (11)	*S. aureus*—Tetracycline
0% (49)	0% (32)	0% (16)	0% (10)	0% (11)	*S. aureus*—TMP-SMX
42% (24)	57% (7)	NT	100% (1)	33% (3)	*E. coli*—Ampicillin
12% (25)	14% (7)	NT	0% (1)	0% (3)	*E. coli*—Amoxicillin–clavulanate
12% (25)	29% (7)	NT	100% (1)	33% (3)	*E. coli*—Ciprofloxacin
17% (24)	29% (7)	NT	0% (1)	0% (3)	*E. coli*—TMP-SMX
60% (20)	90% (10)	50% (2)	NT	0% (1)	*P. mirabilis*—Ampicillin
35% (20)	43% (14)	50% (2)	NT	0% (1)	*P. mirabilis*—Ciprofloxacin
70% (20)	64% (14)	50% (2)	NT	0% (1)	*P. mirabilis*—TMP-SMX
5% (20)	7% (14)	50% (2)	NT	NT	*P. mirabilis*—Cefuroxime
50% (2)	0% (2)	0% (1)	NT	NT	*P. mirabilis*—Meropenem
10% (10)	18% (11)	0% (3)	0% (5)	0% (4)	*P. aeruginosa*—Ciprofloxacin
11% (9)	9% (11)	0% (3)	20% (5)	0% (4)	*P. aeruginosa*—Ceftazidime
20% (10)	9% (11)	0% (3)	0% (1)	33% (3)	*P. aeruginosa*—Pip–tazobactam
24% (75)	36% (44)	69% (13)	0% (1)	100% (2)	Enterobacterales—Cefuroxime
11% (18)	0% (9)	0% (10)	NT	NT	Enterobacterales—Carbapenem
0% (14)	0% (9)	0% (2)	NT	0% (4)	*E. faecalis*—Ampicillin
0% (12)	0% (9)	0% (2)	NT	NT	*E. faecalis*—Vancomycin

**Table 4:** Clinical characteristics and outcomes of patients with *Enterococcus faecalis*-positive diabetic foot infection, 2021–2025. Denominators vary because not all variables were recorded for every patient. ABI, ankle–brachial index.

Value (n = 29)	Characteristic
66 (50–85)	Age, median (range), years
23	Male sex, n
23/28	Polymicrobial culture (>1 organism), n/N
2 (1–4)	Pathogens per culture, median (range)
19/28	Peripheral arterial disease (ABI < 0.9), n/N
14/28	Peripheral neuropathy, n/N
3	Immunocompromise, n
20/28	University of Texas wound grade 3 (bone/joint), n/N
17/28	Fever at presentation, n/N
29 (100%)	Ampicillin-susceptible, n
100%	Vancomycin/teicoplanin-susceptible (where tested)
15	Healed, n
15 (5–30)	Time to healing, median (range), weeks
8	Amputation (minor or major), n
2	Death, n (including 1 with amputation)
1	Treatment ongoing, n
3	Lost to follow-up, n

## Data Availability

The de-identified isolate-level dataset analysed during the current study is available from the corresponding author upon reasonable request, subject to institutional data-sharing policy. De-identified patient-level clinical data for the *Enterococcus faecalis* subgroup are provided in Supplementary Table S1.

## Supplementary Materials

The following supporting information can be downloaded at: https://www.mdpi.com/article/10.3390/antibiotics15080723/s1, Table S1: De-identified patient-level clinical characteristics and outcomes of patients with *Enterococcus faecalis*-positive diabetic foot infection, 2021–2025.
